# Evaluation of the humoral immune response and cross reactivity against *Mycobacterium tuberculosis* of mice immunized with liposomes containing glycolipids of *Mycobacterium smegmatis*

**DOI:** 10.1186/1471-2172-14-S1-S13

**Published:** 2013-02-25

**Authors:** Reinier Borrero, María de los A García, Liem Canet, Caridad Zayas, Fátima Reyes, Jorge L Prieto, Juan F Infante, María E Lanio, Ramlah Kadir, Yamilé López, María E Sarmiento, Mohd Nor Norazmi, Armando Acosta

**Affiliations:** 1 Molecular Biology Department, Finlay Institute, La Lisa. La Habana, Cuba, P.O. Box 16017; 2 Center for Protein Study, Faculty of Biology, University of Havana, La Habana, Cuba, ZP 10400; 3 School of Health Sciences, Universiti Sains Malaysia, 16150 Kubang Kerian, Kelantan, Malaysia; 4 Institute for Research in Molecular Medicine, Universiti Sains Malaysia, 16150 Kubang Kerian, Kelantan, Malaysia

## Abstract

*Mycobacterium smegmati*s (Ms) is a nonpathogenic mycobacteria of rapid growth, which shares many characteristics with *Mycobacterium tuberculosis* (MTB), the major causative agent of tuberculosis. MTB has several cell wall glycolipids in common with Ms, which play an important role in the pathogenesis of tuberculosis and the induction of a protective immune response against MTB infection in some animal models. In this study, the humoral immune response and cross reactivity against MTB, of liposomes containing a mixture of cell wall glycolipids of Ms and commercial lipids was evaluated, in order to study its possible use as a component of a vaccine candidate against tuberculosis. Liposomes containing total lipids extracted from Ms, distearoyl phosphatidyl choline and cholesterol were prepared by the dehydration-rehydration technique. Balb/c mice were immunized with the liposomes obtained and the antibody response and cross reactivity against MTB were tested by ELISA. Total lipids extract from Ms showed the presence of several polar glycolipids in common with MTB, such as phosphatidylinositol mannosides. Liposomes that contained glycolipids of Ms were capable of inducing a specific IgG antibody response that allowed the recognition of surface antigens of MTB. The results of this study demonstrated the presence of immunogenic glycolipids in Ms, which could be included to enhance the protective effects of subunit vaccine formulations against tuberculosis.

## Introduction

Tuberculosis (TB) is still one of the most lethal infectious diseases worldwide [1]. The only TB vaccine available for human use is the bacille Calmette-Guerin (BCG), an attenuated strain of *Mycobacterium bovis *[2]. However, this vaccine protects only against the most severe forms of the disease during childhood and their effectiveness has failed in the adult population. In recent years various glycolipids of the mycobacterial cell wall have become attractive candidates for a vaccine against TB because they constitute important virulence factors for initiating the establishment of infection by MTB [3]. These glycolipids have been demonstrated to be capable of stimulating T lymphocytes through the presentation by CD1 molecules [4]. In addition, mycobacterium lipids have been shown to induce protection in certain experimental animal models against infection by MTB [5,6]. Some of these glycolipids are present in the cell wall of Mycobacterium smegmatis (Ms), a non-pathogenic mycobacteria of rapid growth, which has been used as a study model for TB. Liposomes prepared from glycolipids of Ms have also been reported to possess immunoadjuvant capacity [7]. Hence, it is interesting to evaluate the use of cell wall glycolipids of Ms as potential vaccine candidates against TB. Therefore, this study aims to assess the humoral immune response and cross reactivity induced by immunization of mice with liposomes containing a mixture of Ms total lipids and commercial lipids against MTB cell wall antigens.

## Materials and methods

Total lipids from Ms mc^2^ 155 were extracted according to the methodology proposed by Rosenkrands et al [8]. Briefly, 30 mL of chloroform: methanol (2:1) was added to 10 g wet weight of biomass and mixed in a shaker overnight at room temperature. The chloroform phase and the extracted lipids were analyzed by one dimensional TLC. Liposomes were prepared according to the technical procedure of dehydration-rehydration [9]. Lipid composition of liposomes was basically distearoyl phosphatidyl choline (Sigma) and cholesterol (Sigma) at a molar ratio 1:1. Ten mg of Ms total lipids was then added to this mixture. Balb/c mice between 8 to 12 weeks were used in this study. Mice were divided into 4 groups of 10 animals each and were immunized subcutaneously with the appropriate preparation obtained as described below. Two control groups were used: the negative control group received 0.1 mL of PBS and the positive control group recieved 5 x 10^6^ CFU of *M. bovis* BCG Danish strain. Another group received 0.1 mL of a liposomal preparation formulated with commercial lipids (LipE) and a third group was inoculated with 0.1 mL of a liposomal preparation containing 1 mg of Ms total lipids and commercial lipids (LipMs). Groups inoculated with the liposome preparation (LipE and LipMs) received 3 doses administered 21 days apart from each other. The humoral immune response and cross reactivity against MTB was evaluated by indirect ELISA using Ms total lipid and subcellular fraction of MTB cell wall (CW), respectively.

## Results and discussion

**Figure 1:**
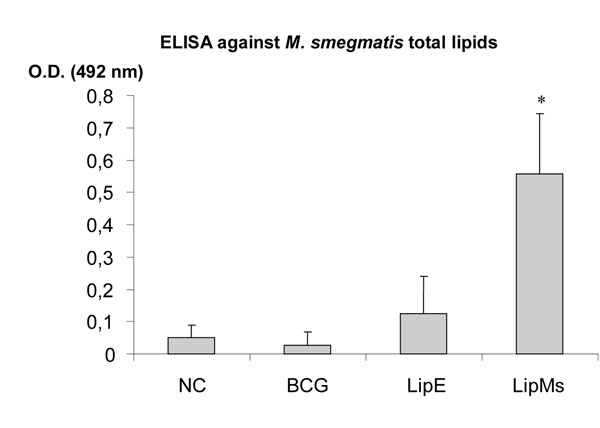
Specific recognition of total lipids of Ms by IgG antibodies present in the sera of mice immunized with liposomes containing lipids of Ms. NC: negative control (PBS), BCG: M. bovis BCG, LipE: liposomes prepared only with commercial lipids, LipMs: liposomes prepared with total lipids of Ms. (*) Significant difference compared to other groups, p <0.05.

**Figure 2:**
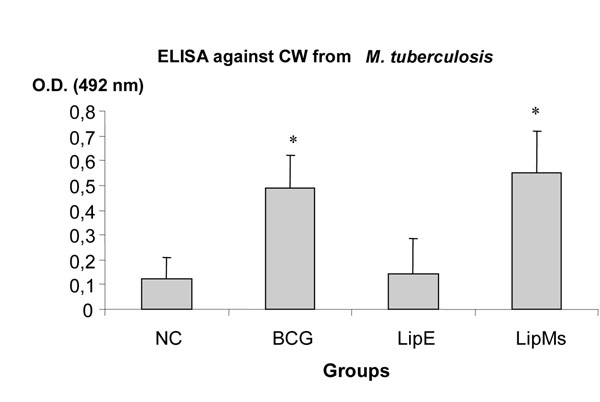
Recognition of MTB antigens by IgG antibodies present in the sera of mice immunized with liposomes containing Ms lipids. NC: negative contol (PBS), BCG: M. bovis BCG, LipE: liposomes prepared only with commercial lipids, LipMs: liposomes prepared with Ms total lipids and commercial lipids. (*) Significant difference compared to the negative groups (NC and LipE), p <0.05.

Recognition of Ms total lipids by IgG antibodies in the sera of animals immunized with LipMs (Figure 1), was significantly higher than that of other groups. However, no significant differences were observed in the sera of animals inoculated with LipE and PBS against lipids from Ms. This result shows that the response obtained with LipMs, is due to the presence of the lipids from Ms and not to the commercial lipids used for preparation of liposomes. IgG antibodies against CW of MTB and BCG was detected in the sera of animals immunized with LipMs (Figure 2). The cross reactivity is possibly due to the presence of some glycolipids in the LipMs that share epitopes in common with MTB and BCG. Moreover, TLC analysis showed the presence of several common polar glycolipids in Ms and MTB like phosphatidylinositol mannosides (PIMs). This lipids have been demonstrated to play an important role in the infection of macrophages and to have the potential to induce humoral immune response capable of conferring protection against infection with MTB in experimental mouse models [10].

In this study, we demonstrated that total lipids of Ms included in liposomes are able to induce humoral immune response in mice and posses cross-reactivity to MTB. These results support the strategy of employing Ms as a possible source of lipid antigens to be part of a vaccine candidate against TB.

## Competing interests

The authors declare that they have no competing financial interests.

## Authors' contributions

All authors have read and approved the final manuscript. RB and MAG were involved in the production of liposomes, animal experiments, evaluation of immunogenicity and cross reactivity, data analyses and writing of the manuscript. MEL and LC were involved in the production of liposomes. JFI, CZ and JLP performed the animal experiments. FR performed the evaluation of immunogenicity and cross reactivity. MES, MNN and AA conceived the study, participated in data analyses and in writing and finalizing of the manuscript. 

## Declarations

This article has been published as part of *BMC Immunology* Volume 14 Supplement 1, 2013: Proceedings from Delivery Systems and Current strategies to drug design. The full contents of the supplement are available online at http://www.biomedcentral.com/bmcimmunol/supplements/14/S1.

## References

[B1] Smith I Mycobacterium tuberculosis Pathogenesis and Molecular Determinants of Virulence Clin. Microbiol. Rev 2003 16 3 463 496 10.1128/CMR.16.3.463-496.2003 12857778 PMC164219

[B2] Lin MY Geluk A Smith SG Stewart AL Friggen AH Franken KL Verduyn MJ van Meijgaarden KE Voskuil MI Dockrell HM Lack of immune responses to *Mycobacterium tuberculosis* DosR regulon proteins following *Mycobacterium bovis* BCG vaccination Infect Immun 2007 75 7 3523 3530 10.1128/IAI.01999-06 17502400 PMC1932964

[B3] Gorocica P Jiménez-Martínez MC Garfias Y Sada I Lascurain R Componentes glicosilados de la envoltura de *Mycobacterium tuberculosis* que intervienen en la patogénesis de la tuberculosis Rev Inst Nal Enf Resp Mex 2005 18 2 142 153

[B4] Mori L De Libero G Presentation of lipid antigens to T cells Immunology Letters 2008 117 1 8 10.1016/j.imlet.2007.11.027 18243339

[B5] Lang ML Glatman-Freedman A Do CD1-Restricted T Cells Contribute to Antibody-Mediated Immunity against *Mycobacterium tuberculosis*? Infect. Immun 2006 74 2 803 809 10.1128/IAI.74.2.803-809.2006 16428722 PMC1360325

[B6] Dascher C Hiromatsu K Xiong X Morehouse C Watts G Llu G McMurray D LeClair K Porcelli S Brenner M Immunization with a mycobacterial lipid vaccine improves pulmonary pathology in the guinea pig model of tuberculosis Int. Immunol 2003 15 8 915 925 10.1093/intimm/dxg091 12882829

[B7] Faisal SM Yan WW McDonough SP Mohamed H Divers T Chang Y Immune response and prophylactic efficacy of Smegmosomes in a hamster model of leptospirosis Vaccine 2009 27 44 6129 6136 10.1016/j.vaccine.2009.08.029 19715780

[B8] Rosenkrands I Agger E Olsen A Korsholm K Swetman C Jensen K Andersen P Cationic Liposomes Containing Mycobacterial Lipids: a New Powerful Th1 Adjuvant System Infect. Immun 2005 73 9 5817 5826 10.1128/IAI.73.9.5817-5826.2005 16113300 PMC1231148

[B9] Perrie Y McNeil S Vangala A Liposome-mediated DNA immunisation via the subcutaneous route J Drug Target 2003 11 8-10 555 563 10.1080/10611860410001670071 15203925

[B10] Singh AP Khuller GK Liposomes as a carrier for mannophosphoinositide antigens of mycobacteria Indian J. Biochem. Biophys 1993 30 160 165 8406546

